# Prevalence of HIV infection among transgender women and travestis in Brazil: data from the TransOdara study

**DOI:** 10.1590/1980-549720240004.supl.1

**Published:** 2024-08-19

**Authors:** Inês Dourado, Laio Magno, Beo Oliveira Leite, Francisco Inácio Bastos, Jurema Corrêa da Mota, Maria Amélia de Sousa Mascena Veras, Marcia Jorge Castejon, TransOdara Research Group

**Affiliations:** I Universidade Federal da Bahia, Institute of Public Health – Salvador (BA), Brazil.; II Universidade do Estado da Bahia, Department of Life Sciences – Salvador (BA), Brazil.; III Fundação Oswaldo Cruz, Institute of Scientific and Technological Communication and Information in Health – Rio de Janeiro (RJ), Brazil.; IV Santa Casa de São Paulo, School of Medical Sciences – São Paulo (SP), Brazil.; V Instituto Adolfo Lutz, Centro de Imunologia - São Paulo (SP), Brazil.

**Keywords:** HIV, Travestis, Transgender persons, Prevalence, Brazil.

## Abstract

**Objective:**

The aim of this study was to investigate the prevalence of human immunodeficiency virus (HIV) infection among transgender women and *travestis* and to analyze factors associated with HIV infection in Brazil.

**Methods:**

TransOdara was a cross-sectional study on sexually transmitted infections among transgender women and *travestis* in five Brazilian cities between 2019 and 2021. Self-identified transgender women and *travestis* aged ≥18 years were recruited using respondent-driven sampling, completed an interviewer-led questionnaire, and provided samples to detect HIV. The outcome was the result of the rapid antigen testing for HIV. Adjusted prevalence ratios (aPR) and 95% confidence intervals (95% CI) were obtained using Poisson regression with robust variance.

**Results:**

Overall, this population was found to be especially vulnerable, with high levels of unstable housing and engagement in informal work. They usually resort to transactional sex as their main working activity. Half of them earned less than the Brazilian minimum wage, which characterizes a poor population living in dire conditions. The overall HIV prevalence was 34.40%. In the final model, the variables associated with the HIV prevalence were as follows: to be 31 years old or older, not studying at the moment they were interviewed, to be unemployed, and engaged in lifetime transactional sex.

**Conclusion:**

We found disproportionately high HIV prevalence among transgender women and *travestis*, compared with a low prevalence among respective segments of Brazil’s general population, which highlights the context of vulnerability in this population. The data point to the urgency for intensification and expansion of access to HIV prevention and strategies to stop discrimination in health care (among other services and contexts) and provide comprehensive services for this population.

## INTRODUCTION

The health of transgender women has been increasingly a subject of research^1^, given the urgent health needs of this population^2-6^. Global estimates of human immunodeficiency virus (HIV) prevalence among transgender women for a pool of several countries were 19.9% in the first decade of the 21st century, corresponding to an odds ratio (OR) of 66.0 when it is cross-compared with adults of reproductive age from the general population^7^. The pronounced lack of any proportionality between infection rates and demographic data is made evident when cross-comparisons between transgender women and the general population are carried out, whatever the geographic unit under analysis, worldwide^8-12^.

Recent data highlight the unique health needs of transgender women, in addition to disproportionately higher rates of HIV infection^7,13^ and syphilis^14^. A global systematic review reported an overall prevalence of HIV infection of 19.9% among transgender women^7^. The estimated prevalence for a set of Brazilian studies was even higher — 14.3% (95%CI 6.8–21.8) to 40.9% (95%CI 35.7–46.1) in 2021^7^. However, there are few studies that estimate the prevalence of HIV among transgender women and *travestis* in Brazil. In general, the prevalence is high but varies according to the region and the methodology used by the studies. Point prevalence varied from 12 to 32% in serosurveys carried out in 2013 and 2017, respectively^11,12,15,16^, much higher when compared to the general Brazilian female population in 2015 (0.4%)^17^.

Such inequities regarding HIV and sexually transmitted infections (STIs) can be explained by contexts of vulnerability that contribute to increased risk for HIV and other STIs: structural venerability includes poor socioeconomic conditions (e.g., when gender identity represents a barrier to ensuring stable employment and, in many contexts, housing) and difficult access to prevention and care services for HIV and other STIs (e.g., discrimination in health services by health professionals or users and a lack of qualified care)^18-21^; interpersonal vulnerability, such as stigma, discrimination, and violence driven by gender identity (e.g., transphobia), within social interactions creates further vulnerability to HIV^22-25^. The biological and psychosocial vulnerability dimensions are composed of sexual behaviors and practices^24,26-29^.

Condomless sex between transgender women and their sexual partners is the key proximal risk factor for HIV infection^29^. The broad picture of entangled risks comprises transactional sex^8,9,30-34^, multiple partners^8,9,31,34^ and the misuse of substances, especially immediately before or during sex^31,32,34^, altering the capacity of individuals to adopt and maintain safer sexual behaviors^35^, besides the obvious risks associated with the shared use of injection paraphernalia.

Epidemiological studies about HIV infection among transgender women in Brazil are relatively rare and are geographically clustered in a few places in the southeast, the southern part of the northeastern region, and, to a lesser extent, in the south^7,36^. They are practically absent in the vast north and center-west regions. They do exist, but a tiny fraction of them are population-based studies of transgender women in context, besides those carried out in referral services for the prevention and treatment of this population, based on convenience samples. We aimed to investigate the regional prevalence of HIV infection among transgender women and *travestis* in Brazil and analyze factors associated with HIV infection.

## METHODS

This is an analysis of transgender women and *travestis* data collected in five capital cities (Campo Grande, Manaus, Porto Alegre, Salvador, and São Paulo) located in all five Brazilian macro-regions, from December 2019 to July 2021, that composed the TransOdara study, a survey aimed at estimating the prevalence of HIV and other STIs and monitoring risk practices for these infections.

“Transgender women and *travestis*” is used here as an umbrella term that includes all individuals who self-identify with a gender identity different than the male sex assigned at birth.

Participants were recruited using respondent-driven sampling (RDS), a chain-link sampling method that begins with “seeds” — a convenience sample of members of the target population chosen by the researchers^37^. In RDS, participants recruited their acquaintances using a coupon system^37^. A maximum of three recruiters were allowed per participant to reduce recruitment homophily. The eligibility criteria were to be 18 years of age or older, to identify with a feminine gender identity at the time of study, to have been assigned male sex at birth, and to spend most of the day in the studied municipalities. They also had to present a valid invitation coupon, agree to participate in the study, and sign the informed consent form. Transgender women and *travestis*, who when interviewed, were under the effect or influence of drugs, including alcohol, in a manner that rendered it difficult for them to understand the research, were excluded.

The researchers selected the first participants, the so-called seeds, after qualitative formative research to represent the heterogeneity of the transgender women and *travestis* population according to demographic and socioeconomic conditions. In each city, 5–10 seeds launched the recruitment process. Each seed, and later each participant, received three coupons to invite transgender women and *travestis* from their social contact network (referral chains). For successful recruitment, RDS includes primary and secondary incentives. The primary one was US$10.00 as compensation for transportation and lost work time. The secondary one, as compensation for the recruitment of contacts, was US$10.00 for each transgender women and *travestis* recruited for the study. All completed a standard investigator-led questionnaire for sociodemographic information and sexual behavior, in a space reserved exclusively for this purpose. Further details are found in Veras et al.^38^

### Study variables

The outcome variable was the result of the rapid antigen HIV test, classified as positive or negative. Other study variables used in this study were gender identity (transgender women and *travestis versus travesti versus* agender/other female identification), sexual orientation (heterosexual versus bisexual/pansexual/homosexual/gay/lesbian/asexual/other) age (18–30 years *versus* 31–68 years), self-reported race/skin color (whites *versus* blacks/brown), studying at the moment they were interviewed (yes *versus* no); schooling (no education/only primary-level *versus* secondary-level education *versus* higher education); living situation (stable: own house or apartment, rented house or apartment *versus* unstable: family and friends, street situation; shelter or boarding house); occupation (employee with a formal contract *versus* employee without a formal contract *versus* unemployed); minimum wage in Brazilian reais (one or less *versus* more than one); and lifetime transactional sex work (no *versus* yes).

### Data analysis

The sociodemographics of the study population were described according to the results of the HIV test. The associations between the study variables and HIV prevalence were measured by the prevalence ratio (PR) with the respective 95% CI in bivariate and multivariate analyses using a mixed Poisson regression with robust variance (a useful tutorial for beginners using R is available at https://stats.oarc.ucla.edu/r/dae/poisson-regression/).

Random intercepts have been applied to each one of the sites (i.e. the respective cities) included in the multicenter study. The choice of using random intercepts is secondary to the between-site heterogeneity, both respecting HIV background infection rates and the sociodemographic characteristics of each site.

Using the abovementioned model, we assumed that we could capture (at least partially) the effects secondary to the different heterogeneities. We took into consideration the American Statistical Association (ASA) statement^39^, i.e. considering that every statistical test should be understood in the context of its application, analytic procedures, and purpose. Weighting was not included in the analyses as suggested by Sperandei et al.^40^, respecting the differential accuracy of RDS indicators for underlying networks with varying natures.

The variables with a p-value≤0.20 in the bivariate analysis were selected to start modeling, and those with a p-value<0.05 remained in the final model, along with variables that were theoretically important, using the backward approach. The adequacy of the final models was analyzed using the Akaike information criteria. All analyses were conducted using R version 4.2.0^41^, comprising libraries lme4 and statistical models.

### Ethical aspects

The project was approved by the Research Ethics Committee of the Santa Casa de Misericórdia de São Paulo (CAAE 05585518.7.0000.5479; opinion n°: 3.126.815 – 30/01/2019), as well as by other participating institutions: Instituto Leônidas and Maria Deane-ILMD/Fiocruz – Manaus; STD/AIDS Reference and Training Center, Instituto Adolfo Lutz; Municipal Secretary of Health of Porto Alegre; Federal University of Bahia; Federal University of Mato Grosso do Sul-UFMS, Federal University of Health Sciences of Porto Alegre; and Federal University of Rio Grande do Sul-UFRS. Written informed consent was obtained from all individual participants included in the study. Transgender women and *travestis* were involved in the design and implementation of the study and led the recruitment of participants using RDS.

## RESULTS

Of the 1,317 transgender women and *travestis* recruited and interviewed, 1,282 (97.3%) agreed to be tested for HIV. Among them, the overall prevalence was 34.40% (95%CI 31.8–37.1%) (number of positives=441). According to each city, the estimated prevalence was 26.55% (107) for São Paulo, 27.59% (48) for Campo Grande, 31.46% (56) for Salvador, 36.69% (124) for Manaus, and 56.08% (106) for Porto Alegre.

Most interviewees self-defined themselves as women or transsexual women (66.6%), heterosexual (79.2%), or aged up to 30 years (50.9%). A substantial majority self-defined themselves as Black (45.6%) and were not studying at the moment they were interviewed (78.6%). Slightly above half of them (52.1%) had completed high school, lived in unstable housing conditions (74.2%), have been engaged in informal work (79.7%), and had lifetime transactional sex as their main working activity (73.7%). Half (50.9%) earned less than the Brazilian minimum wage per month (Table 1).

**Table 1:** Bivariate analysis of factors associated with HIV infection among TransOdara participants (December 2019–July 2021).

Variables	Total	HIV			PR (95%CI)
	n (%)	Negative (n=841)	Positive (n=441)	% (34.40)	
Sites					
Campo Grande	174 (13.6)	126	48	27.59	
Manaus	338 (26.4)	213	124	36.69	
Porto Alegre	189 (14.7)	83	106	56.08	
Salvador	178 (13.9)	122	56	31.46	
São Paulo	403 (31.4)	296	107	26.55	
Gender identities					
Agender/Other female identification	44 (3.4)	36	8	18.18	1.00
Transsexual women	852 (66.6)	580	272	31.92	1.77 (0.87–3.58)
*Travesti*	384 (30.0)	224	160	41.67	2.27 (1.11–4.63)
Sexual orientations					
Heterosexual	1006 (79.2)	642	364	36.18	1.43 (1.10–1.85)
Bisexual/Pansexual/Homosexual/Gay/Lesbian/Asexual/Other	264 (20.8)	191	73	27.65	1.00
Age (years)					
18–30	652 (50.9)	497	155	23.77	1.00
31–68	630 (49.1)	344	286	45.40	1.93 (1.58–2.35)
Race/skin color					
White	330 (26.8)	224	106	32.12	1.00
Brown	339 (27.6)	206	133	39.23	1.29 (0.99–1.67)
Black	560 (45.6)	378	182	32.5	1.13 (0.88–1.45)
Studying at the moment they were interviewed					
Yes	274 (21.4)	206	68	24.82	1.00
No	1005 (78.6)	634	371	36.92	1.49 (1.15–1.93)
Schooling					
No education/only primary-level education	547 (42.8)	319	228	41.68	1.61 (0.98–2.64)
Secondary-level education	667 (52.1)	474	193	28.94	1.13 (0.69–1.85)
Higher education	65 (5.1)	48	17	26.15	1.00
Living situation					
Stable	331 (25.8)	210	121	36.56	1.00
Unstable	951 (74.2)	631	320	33.65	0.92 (0.74–1.14)
Work situation					
Formal	127 (9.9)	99	28	22.05	1.00
Informal	1017 (79.7)	662	355	34.91	1.51 (1.02–2.22)
Unemployed	132 (10.3)	77	55	41.67	1.80 (1.14–2.86)
Brazilian minimum wage					
Less than 1 MW	580 (50.9)	371	209	36.03	1.09 (0.89–1.34)
1 MW or more	560 (49.1)	373	187	33.39	1.00
Lifetime transactional sex work					
No	336 (26.3)	268	68	20.24	1.00
Yes	942 (73.7)	572	370	39.28	1.97 (1.52–2.55)

In the bivariate analyses, the key variables associated with HIV prevalence were found to identify as a *travesti* (PR: 2.27; 95%CI 1.11–4.63), to report heterosexual as sexual orientation (PR: 1.43; 95%CI 1.10–1.85), not studying at the moment they were interviewed (PR: 1.49; 95%CI 1.15–1.93), to be engaged in informal work (PR: 1.51; 95%CI 1.02–2.22) or unemployed (PR: 1.80; 95%CI 1.14–2.86), as well as to be engaged in lifetime transactional sex (PR: 1.97; 95%CI 1.52–2.55) (Table 1).

In the final multivariate model, the variables independently associated with the outcome (HIV prevalence) were the ones as follows: to be 31 years old or more (PR: 1.76; 95%CI 1.44–2.16), not studying at the moment they were interviewed (1.32; 95%CI 1.01–1.73), to be unemployed (1.76; 95%CI 1.10–2.80), and to be engaged in lifetime transactional sex (1.76; 95%CI 1.35–2.29) (Table 2).

**Table 2:** Multivariate analysis of factors associated with HIV infection among TransOdara participants (December 2019–July 2021).

Variables	aPR	95%CI
Age: 31–68 years	1.76	[1.44–2.16]
Studying at the moment they were interviewed: No	1.32	[1.01–1.73]
Work situation: Informal	1.43	[0.97–2.12]
Work situation: Unemployed	1.76	[1.10–2.80]
Lifetime transactional sex: Yes	1.76	[1.35–2.29]

aPR: adjusted prevalence ratio.

Cross-comparative performance using fixed components models.

Model based on the statistical significance of bivariate analyses: gender identity + sexual orientation + age group + studying [in the moment of the interview] + engaged in the working force + lifetime transactional sex.

Final model based on the model with the best fit.

AIC = 1694.7; BIC = 1746.1; Deviance = 1674.7.

## DISCUSSION

This study has made evident a very high pooled prevalence for HIV among the large number of transgender women and *travestis* interviewed, as well as a high point prevalence for each one of the sites included in the multicenter study, ranging from 26.32 to 56.08%.

The results corroborate previous findings from both population-based studies as well as studies with convenience samples from referral centers^7^. Whatever the setting and the sampling strategy, HIV infection rates are always very high and much higher than those observed among women in the general population^7^.

One of the hypotheses for the much higher rate in Porto Alegre is the specificity of the epidemic in Rio Grande do Sul (RS). The acquired immune deficiency syndrome (AIDS) detection rate in RS (24.3 per 100,000 inhabitants) in 2022 was one and a half times higher than that for the country (16.5 per 100,000 inhabitants). The mortality rate was also higher (7.7 deaths per 100,000 inhabitants) in RS than the national average, which was 4.2 deaths per 100,000 inhabitants. The highest mortality rate was estimated for Porto Alegre (22.6 deaths per 100,000 inhabitants), five times the national rate. According to the composite index based on the indicators of AIDS detection rate, mortality rate, and first CD4 cell count in the past five years, Porto Alegre ranked in the second highest position among the Brazilian capitals^42^.

In a population deeply affected by stigma and marginalization^6,23,43^, living in dire conditions characterized by unstable housing^43-45^, enormous difficulties engaging in the formal labor market, low income, and frequent unemployment^46-48^, this inauspicious finding is by no means surprising. The several adverse conditions and risk factors tend to cluster and frequently interact, all of them contributing to unacceptable living standards and high vulnerability to HIV (among other infections and other medical and social conditions not assessed in the current study).

In Brazil, HIV prevalence in this population is very high and seems unlikely to be curbed or even substantially ameliorated, with the exception of women who benefit from state-of-the-art prevention programs, including pre-exposure prophylaxis (PrEP)^49-52^.

A systematic review and meta-analysis of Brazilian studies implemented between 1995 and 2016 made evident prevalence rates varying between 14.3% (6.8–21.8) and 40.9 (35.7–46.1)^7^. More recent studies have shown point prevalence’s slightly lower, despite the fact that confidence intervals still tend to overlap, varying from 9.0% (4.2–18.2) to 31.2% (18.8–43.6) for studies carried out in Salvador^53^ and Rio de Janeiro^15^, respectively.

Since sampling errors tend to be ignored (in the case of convenience samples) or are very hard to estimate (in the case of studies using the available alternatives to probability studies, such as RDS), it is difficult to distinguish trends toward a putative decline of infection rates in recent years from biases secondary to statistical inference in non-probability samples^54^.

High prevalence rates have also been shown in studies in Argentina (34.1%)^55^ and Uruguay (21.5%)^8^. Of much concern is the fact that prevalence rates are almost invariably higher than the single pooled estimate for this population worldwide (point prevalence of 19.9%)^7^.

In Brazil and in the vast majority of countries and specific settings, transgender women live in contexts of high vulnerability and face stigmatization and marginalization on a daily basis^5,23,56-58^. Brazil remains the country where violence against transgender women is more prevalent, with a very high level of homicides (vis-à-vis the relatively small denominators)^59,60^. Despite the undeniable gains of targeted projects, this unfortunate situation calls for structural changes, with the deep commitment of the government at different levels and civil society.

A comprehensive set of coordinated initiatives is sorely needed, combining targeted interventions (e.g., in the field of health) with structural changes, increasing the chances that this population may have full access to education, employment, and working opportunities in different types of jobs, especially those currently far from accessible, i.e., well-paid, high-qualified jobs^61-65^. The high rates of engagement in the informal market, the high rates of unemployment, and the very low income of the vast majority of these women show such goals remain a dream rather than concrete opportunities.

A deep gap remains between what has been promised and has been several times translated into non-discriminatory laws and initiatives. Of course, such legal benchmarks are essential, but they should be translated into practical and continuous initiatives. Prejudice is deeply entrenched and pervasive. The fight to curb it must be as present and relentless as its open and disguised roots.

It is not clear whether HIV infection rates are really decreasing in recent years or that the putative trends may be just a consequence of many gaps, caveats, and biases. Whatever the truth, the real or perceived decrease is too slow and will cost the lives of several people. People have already been victimized by homicides and other severe medical conditions. These figures are not compatible with what we could call a humane and compassionate society.

This study has limitations. The cross-sectional design poses challenges in capturing temporal relationships within the explored associations. The RDS methodology’s design may introduce selection bias through non-probabilistic sampling and network homophily, though it does not rule out the feasibility of conducting such inquiries in hard-to-reach populations. To minimize drawing bias, statistical analyses incorporated the city as a random intercept in the mixed-effects Poisson regression models. Nevertheless, these limitations do not preclude us from gathering valuable information about the contact networks of our sample, aligning with other findings in the literature.

Despite Brazil making progress with public policies (e.g., the National Policy on STIs/AIDS and the National Policy on LGBT Integral Health), many barriers remain, consequently impeding the full implementation of HIV prevention strategies. Gender discrimination has been described in the literature as a structuring factor of social inequities for the transgender population, particularly in increasing the vulnerability of transgender women and *travestis* to HIV infection. This study reinforces the need to prioritize the right to dignified social well-being and quality of life, as well as clearly indicating the urgency to intensify and expand access to HIV prevention and the implementation of strategies that disrupt the discrimination experienced in healthcare services (among others) and provide appropriate services for this population.
