# Erratum

**DOI:** 10.1590/1980-549720240004.supl.1erratum

**Published:** 2024-10-21

In the manuscript **"Prevalence of HIV infection among transgender women and travestis in Brazil: data from the TransOdara study"**, DOI: https://doi.org/10.1590/1980-549720240004.supl.1, published in the Rev Bras Epidemiol. 2024; 27(Suppl 1): e240004.supl.1

**Where it reads:**

https://doi.org/10.1590/1980-549720230004.supl.1

**It should read:**

https://doi.org/10.1590/1980-549720240004.supl.1

**On page 1 it was included:**

Marcia Jorge Castejon^V^ http://orcid.org/0000-0002-3525-740X

^V^Instituto Adolfo Lutz, Centro de Imunologia - São Paulo, SP – Brasil.

**On page 9, where it reads:**

**AUTHORS’ CONTRIBUTIONS:** ID: conceptualization, data curation, formal analysis, investigation, methodology, supervision, writing – original draft, writing – review & editing. BOL: data curation, formal analysis, investigation, methodology, writing – original draft, writing – review & editing. LM: conceptualization, data curation, formal analysis, investigation, methodology, supervision, writing – original draft, writing – review & editing. FIB: conceptualization, investigation, methodology, writing – original draft, writing – review & editing. JCM: data curation, formal analysis, methodology, writing – original draft, writing – review & editing. MASMV: conceptualization, data curation, formal analysis, funding acquisition, investigation, methodology, project administration, supervision, writing – original draft; writing – review & editing.

**It should read:**

**AUTHORS’ CONTRIBUTIONS:** ID: conceptualization, data curation, formal analysis, investigation, methodology, supervision, writing – original draft, writing – review & editing. BOL: data curation, formal analysis, investigation, methodology, writing – original draft, writing – review & editing. LM: conceptualization, data curation, formal analysis, investigation, methodology, supervision, writing – original draft, writing – review & editing. FIB: conceptualization, investigation, methodology, writing – original draft, writing – review & editing. MJC: data curation, supervision, formal analysis, writing – review & editing. JCM: data curation, formal analysis, methodology, writing – original draft, writing – review & editing. MASMV: conceptualization, data curation, formal analysis, funding acquisition, investigation, methodology, project administration, supervision, writing – original draft; writing – review & editing.

