# Supplementary material for: Prevalence of HIV infection among transgender women and travestis in Brazil: data from the TransOdara study
Source: Rev Bras Epidemiol. 2024 Aug 19;27(Suppl 1):e240004.supl.1. doi: 10.1590/1980-549720240004.supl.1 (PMC11495135; doi:10.1590/1980-549720240004.supl.1)
Supplement: Translation

# Translation: Prevalência de infecção pelo vírus da imunodeficiência humana (HIV) em mulheres trans e travestis no Brasil: dados do estudo TransOdara

**DOI:** 10.1590/1980-549720240004.supl.1.2

**Authors:** Inês Dourado, Laio Magno, Beo Oliveira Leite, Francisco Inácio Bastos, Jurema Corrêa da Mota, Maria Amélia de Sousa Mascena Veras, Marcia Jorge Castejon, TransOdara Research Group

**Affiliations:** I Universidade Federal da Bahia, Instituto de Saúde Coletiva – Salvador (BA), Brasil.; II Universidade do Estado da Bahia, Departamento de Ciências da Vida – Salvador (BA), Brasil.; III Fundação Oswaldo Cruz, Instituto de Comunicação e Informação Científica e Tecnológica em Saúde, Laboratório de Informações em Saúde – Rio de Janeiro (RJ), Brasil.; IV Santa Casa de São Paulo, Faculdade de Ciências Médicas – São Paulo (SP), Brasil.; V Instituto Adolfo Lutz, Centro de Imunologia - São Paulo (SP), Brasil.

**Keywords:** HIV, Travestis, Pessoas transsexuais, Prevalência, Brasil

## Abstract

**Objetivo:**

Investigar a prevalência do vírus da imunodeficiência humana (HIV) entre mulheres trans e travestis em cinco capitais no Brasil.

**Métodos:**

TransOdara foi um estudo de corte transversal que avaliou comportamentos e a prevalência de infecções sexualmente transmissíveis (IST) entre mulheres trans e travestis em cinco capitais brasileiras, entre 2019 e 2021. Mulheres trans e travestis ≥18 anos foram recrutadas utilizando *respondent-driven sampling*, responderam a um questionário e fizeram testes rápidos para HIV e outras IST. O desfecho foi o resultado do teste rápido para HIV. Estimaram-se razões de prevalência ajustadas e intervalos de confiança de 95% por meio da regressão de Poisson com variância robusta.

**Resultados:**

No seu conjunto, esta população mostrou-se vulnerabilizada, com níveis elevados de moradia instável e no mercado de trabalho informal. Habitualmente, essas mulheres recorrem ao sexo comercial como sua atividade profissional principal. Metade delas receberam menos do que um salário mínimo, vivendo em condições desfavoráveis. A prevalência da infecção pelo HIV foi de 34.40%. No modelo final, as variáveis associadas com a prevalência do HIV foram: ter 31 anos ou mais, não estar estudando no momento da entrevista, estar desempregada e estar engajada em sexo comercial.

**Conclusão:**

Foi identificada uma prevalência substancialmente elevada para o HIV entre as mulheres trans e travestis (em contraste com a prevalência baixa na população geral de mulheres brasileiras), sublinhando o contexto de vulnerabilidade desta população. Os achados indicam claramente a premência de intensificar e expandir o acesso à prevenção do HIV e da implementação de estratégias que interrompam o processo de discriminação vivenciado nos serviços de saúde e ofereçam serviços apropriados a esta população.

## INTRODUÇÃO

A saúde das mulheres trans tem sido cada vez mais objeto de investigação^1^, dadas as urgentes necessidades de saúde dessa população^2-6^. As estimativas globais da prevalência do vírus da imunodeficiência humana (HIV) entre mulheres trans para vários países foram de 19,9% na primeira década do século 21, correspondendo a um *odds ratio* (OR) de 66,0 em comparação com adultos de idade reprodutiva da população em geral^7^. A desproporcionalidade entre as taxas de infecção e os dados demográficos fica evidente quando são realizadas comparações cruzadas entre mulheres trans e a população em geral, qualquer que seja a unidade geográfica em análise, em todo o mundo^8-12^.

Dados recentes destacam as necessidades de saúde de mulheres trans, além de taxas desproporcionalmente mais elevadas de infecção pelo HIV^7,13^ e sífilis^14^. Uma revisão sistemática relatou prevalência global de infecção pelo HIV de 19,9% entre mulheres trans^7^. A prevalência estimada para um conjunto de estudos brasileiros foi ainda maior — 14,3% (IC95% 6,8–21,8) a 40,9% (IC95% 35,7–46,1) em 2021^7^. No entanto, são poucos os estudos que estimam a prevalência do HIV entre mulheres trans e travestis no Brasil. Em geral, a prevalência é elevada, mas varia de acordo com a região e a metodologia utilizada pelos estudos. A prevalência pontual variou de 12 a 32% nos inquéritos sorológicos realizados em 2013 e 2017, respectivamente^11,12,15,16^, muito maior quando comparada à população geral feminina brasileira em 2015 (0,4%)^17^.

Tais desigualdades em relação ao HIV e às infecções sexualmente transmissíveis (IST) podem ser explicadas por contextos de vulnerabilidade que contribuem para o aumento do risco de HIV e outras IST: a vulnerabilidade estrutural inclui más condições socioeconômicas (por exemplo, quando a identidade de gênero representa uma barreira para garantir um emprego estável e, em muitos contextos, moradia) e difícil acesso a serviços de prevenção e cuidados para o HIV e outras IST (por exemplo, discriminação nos serviços de saúde por parte de profissionais de saúde ou usuários e falta de cuidados qualificados)^18-21^; a vulnerabilidade interpessoal, como o estigma, a discriminação e a violência motivada pela identidade de gênero (por exemplo, transfobia), nas interações sociais cria uma maior vulnerabilidade ao HIV^22-25^. As dimensões da vulnerabilidade biológica e psicossocial são compostas por comportamentos e práticas sexuais^24,26-29^.

O sexo sem preservativo entre mulheres trans e os seus parceiros sexuais é o principal fator de risco proximal para a infecção pelo HIV^29^. A ampla gama de riscos associados compreende o sexo transacional_8,9,30-34_, múltiplos parceiros^8,9,31,34^ e o uso indevido de substâncias, especialmente imediatamente antes ou durante o sexo^31,32,34^, alterando a capacidade dos indivíduos de adotar e manter comportamentos sexuais mais seguros^35^, além dos riscos óbvios associados ao uso compartilhado de instrumentos para injeção.

Estudos epidemiológicos sobre a infecção pelo HIV entre mulheres trans no Brasil são relativamente raros e estão geograficamente agrupados em alguns locais do Sudeste, na parte sul da região Nordeste e, em menor proporção, no Sul^7,36^. Eles são praticamente inexistentes nas regiões Norte e Centro-Oeste, embora exista uma pequena fração de estudos de base populacional de mulheres trans, além daqueles realizados em serviços de referência para prevenção e tratamento dessa população, a partir de amostras de conveniência. O objetivo deste estudo foi investigar a prevalência da infecção pelo HIV entre mulheres trans e travestis residentes em cinco capitais no Brasil e analisar os fatores associados à infecção pelo HIV.

## METODOLOGIA

Trata-se de uma análise de dados de mulheres trans e travestis coletados em cinco capitais (Campo Grande, Manaus, Porto Alegre, Salvador e São Paulo) localizadas nas cinco macrorregiões brasileiras, no período de dezembro de 2019 a julho de 2021, que compuseram o estudo TransOdara, um inquérito com o objetivo de estimar a prevalência de HIV e de outras IST, e a monitorar práticas de risco para essas infecções.

“Mulheres trans e travestis” é usado aqui como um termo abrangente que inclui todos os indivíduos que se identificam com uma identidade de gênero diferente do sexo masculino atribuído no nascimento.

As participantes foram recrutadas por meio de amostragem orientada por respondentes (*respondent-driven sampling* – RDS), um método de amostragem em cadeia que começa com “sementes” — uma amostra de conveniência de membros da população-alvo escolhida pelos pesquisadores^37^. No RDS, os participantes recrutaram seus conhecidos por meio de um sistema de cupons^37^. Foram permitidos no máximo três recrutadoras por participante para reduzir a homofilia no recrutamento. Os critérios de elegibilidade foram ter idade igual ou superior a 18 anos, identificar-se com identidade de gênero feminina no momento do estudo, ser designada do sexo masculino ao nascer, e passar a maior parte do dia nos municípios estudados. Também era necessária a apresentação de um cupom de convite válido, concordar em participar do estudo e assinar o termo de consentimento livre e esclarecido. Foram excluídas mulheres trans e travestis que, quando entrevistadas, estivessem sob efeito ou influência de drogas, inclusive álcool, de forma que dificultasse a compreensão da pesquisa.

Os pesquisadores selecionaram as primeiras participantes, as chamadas sementes, após pesquisa formativa qualitativa de forma a representar a heterogeneidade da população de mulheres trans e travestis segundo condições demográficas e socioeconômicas. Em cada cidade, 5 a 10 sementes iniciaram o processo de recrutamento. Cada semente, e posteriormente cada participante, recebeu três cupons para convidar mulheres trans e travestis de sua rede social de contato (cadeias de referência). Para um recrutamento bem-sucedido, o RDS incluiu incentivos primários e secundários. O primário foi de US$ 10,00 como indenização por transporte e perda de jornada de trabalho. Além disso, a compensação pelo recrutamento de contatos foi de US$ 10,00 para cada mulher trans e travesti recrutada para o estudo. Todas preencheram um questionário padrão conduzido pelo investigador para coleta de informações sociodemográficas e comportamento sexual, em um espaço reservado exclusivamente para esse fim. Maiores detalhes podem ser encontrados em Veras et al.^38^

### Variáveis do estudo

A variável de desfecho foi o resultado do teste rápido de antígeno do HIV, classificado como positivo ou negativo. Outras variáveis de estudo selecionadas foram a identidade de gênero (mulher trans *versus* travesti *versus* sem gênero/outra identificação feminina), a orientação sexual (heterossexual *versus* bissexual/pansexual/homossexual/gay/lésbica/assexual/outra), a idade (18–30 anos *versus* 31–68 anos), a raça/cor da pele autorreferida (brancos *versus* pretos/pardos), se estava estudando no momento da entrevista (sim *versus* não); a escolaridade (sem escolaridade/apenas ensino fundamental *versus* ensino médio *versus* ensino superior); a situação de moradia (estável: casa ou apartamento próprio, casa ou apartamento alugado *versus* instável: família e amigos, situação de rua; abrigo ou pensão); a ocupação (empregado com carteira assinada *versus* empregado sem carteira assinada *versus* desempregado); o salário mínimo em reais (um ou menos *versus* mais de um); e o trabalho sexual ao longo da vida (não *versus* sim).

### Análise de dados

Os dados sociodemográficos da população estudada foram descritos de acordo com o resultado do teste de HIV. As associações entre as variáveis do estudo, e a prevalência de HIV foram medidas pela razão de prevalência (RP) com os respectivos intervalos de confiança de 95% (IC95%), em análise bivariada. E multivariada utilizando uma regressão de Poisson mista com variância robusta (um tutorial útil para iniciantes que usam R está disponível em https://stats.oarc.ucla.edu/r/dae/poisson-regression/).

Interceptos aleatórios foram aplicados a cada um dos locais (ou seja, as respectivas cidades) incluídos no estudo multicêntrico. A escolha de utilizar interceptos aleatório é subsequente à heterogeneidade entre os locais, respeitando tanto as taxas de infecção pelo HIV basais, como as características sociodemográficas de cada local.

Utilizando o modelo mencionado acima, assumimos que poderíamos capturar (pelo menos parcialmente) os efeitos subsequentes às diferentes heterogeneidades. Levamos em consideração a afirmação da *American Statistical Association* (ASA)^39^, ou seja, consideramos que todo teste estatístico deve ser entendido no contexto de sua aplicação, procedimentos analíticos e finalidade. A ponderação não foi incluída nas análises conforme sugerido por Sperandei et al.^40^, respeitando a acurácia diferencial dos indicadores RDS para redes subjacentes de naturezas variadas.

As variáveis com valor de p≤0,20 na análise bivariada foram selecionadas para inclusão na modelagem inicial, e aquelas com valor de p<0,05 permaneceram no modelo final, juntamente com as variáveis que eram teoricamente importantes, utilizando a abordagem retrospectiva de seleção de variáveis. A adequação dos modelos finais foi analisada através dos critérios de informação de Akaike. Todas as análises foram realizadas no R versão 4.2.041, composto pelas bibliotecas lme4 e modelos estatísticos.

### Aspectos éticos

O projeto foi aprovado pelo Comitê de Ética em Pesquisa (CEP) da Santa Casa de Misericórdia de São Paulo (CAAE 05585518.7.0000.5479 - Nº parecer: 3.126.815 - 30/01/2019), assim como pelas demais instituições participantes: Instituto Leônidas e Maria Deane-ILMD/Fiocruz – Manaus; Centro de Referência e Treinamento em DST/AIDS, Instituto Adolfo Lutz; Secretaria Municipal de Saúde de Porto Alegre; Universidade Federal da Bahia; Universidade Federal de Mato Grosso do Sul-UFMS, Universidade Federal de Ciências da Saúde de Porto Alegre; e Universidade Federal do Rio Grande do Sul-UFRS. O termo de consentimento livre e esclarecido por escrito foi obtido de todos os participantes incluídos no estudo. Mulheres trans e travestis estiveram envolvidas na concepção e implementação do estudo e lideraram o recrutamento de participantes utilizando RDS.

## RESULTADOS

Das 1.317 mulheres trans e travestis recrutadas e entrevistadas, 1.282 (97,3%) concordaram em fazer o teste de HIV. Entre elas, a prevalência geral foi de 34,40% (IC95% 31,8–37,1%) (número de testes positivos=441). Segundo cada cidade, a prevalência estimada foi de 26,55% (107) para São Paulo, 27,59% (48) para Campo Grande, 31,46% (56) para Salvador, 36,69% (124) para Manaus e 56,08% (106) para Porto Alegre.

A maioria das entrevistadas se autodefiniu como mulher ou mulher transexual (66,6%), heterossexual (79,2%), com idade até 30 anos (50,9%). Quase metade se autodefiniu como negra (45,6%) e uma grande maioria não estudava no momento da entrevista (78,6%). Pouco mais da metade (52,1%) completou o ensino médio, vivia em condições de moradia instáveis (74,2%), exercia trabalho informal (79,7%) e referiu trabalho sexual como principal atividade laboral ao longo da vida (73,7%). Metade delas (50,9%) ganhava menos que um salário-mínimo (Tabela 1).

**Tabela 1:** Análise bivariada de fatores associados à infecção pelo HIV entre participantes do TransOdara (dezembro de 2019 a julho de 2021).

Variáveis	Total	HIV			RP (IC95%)
	n (%)	Negativo (n=841)	Positivo (n=441)	% (34,40)	
Locais do estudo					
Campo Grande	174 (13,6)	126	48	27,59	
Manaus	338 (26,4)	213	124	36,69	
Porto Alegre	189 (14,7)	83	106	56,08	
Salvador	178 (13,9)	122	56	31,46	
São Paulo	403 (31,4)	296	107	26,55	
Identidade de gênero					
Sem gênero/Outra identificação feminina	44 (3,4)	36	8	18,18	1,00
Mulheres transexuais	852 (66,6)	580	272	31,92	1,77 (0,87–3,58)
Travesti	384 (30,0)	224	160	41,67	2,27 (1,11–4,63)
Orientação sexuais					
Heterossexual	1006 (79,2)	642	364	36,18	1,43 (1,10–1,85)
Bissexual/Pansexual/Homossexual/Gay/Lésbica/Assexual/Outro	264 (20,8)	191	73	27,65	1,00
Idade (anos)					
18–30	652 (50,9)	497	155	23,77	1,00
31–68	630 (49,1)	344	286	45,40	1,93 (1,58–2,35)
Raça/cor da pele					
Branca	330 (26,8)	224	106	32,12	1,00
Parda	339 (27,6)	206	133	39,23	1,29 (0,99–1,67)
Negra	560 (45,6)	378	182	32,5	1,13 (0,88–1,45)
Estudando no momento em que foram entrevistadas					
Sim	274 (21,4)	206	68	24,82	1,00
Não	1005 (78,6)	634	371	36,92	1,49 (1,15–1,93)
Escolaridade					
Sem escolaridade/apenas educação de nível primário	547 (42,8)	319	228	41,68	1,61 (0,98–2,64)
Educação de nível secundário	667 (52,1)	474	193	28,94	1,13 (0,69–1,85)
Ensino superior	65 (5,1)	48	17	26,15	1,00
Situação de moradia					
Estável	331 (25,8)	210	121	36,56	1,00
Instável	951 (74,2)	631	320	33,65	0,92 (0,74–1,14)
Situação de trabalho					
Formal	127 (9,9)	99	28	22,05	1,00
Informal	1017 (79,7)	662	355	34,91	1,51 (1,02–2,22)
Desempregada	132 (10,3)	77	55	41,67	1,80 (1,14–2,86)
Salário-mínimo					
Menos de 1 SM	580 (50,9)	371	209	36,03	1,09 (0,89–1,34)
1 SM ou mais	560 (49,1)	373	187	33,39	1,00
Trabalho sexual ao longo da vida					
Não	336 (26,3)	268	68	20,24	1,00
Sim	942 (73,7)	572	370	39,28	1,97 (1,52–2,55)

Nas análises bivariadas, as principais variáveis associadas à prevalência do HIV foram identificar-se como travesti (RP: 2,27; IC95% 1,11–4,63), referir-se heterossexual (RP: 1,43; IC95% 1,10–1,85), não estudar no momento da entrevista (RP: 1,49; IC95% 1,15–1,93), exercer trabalho informal (RP: 1,51; IC95% 1,02–2,22) ou desemprego (RP: 1,80; IC95% 1,14–2,86), bem como referência a trabalho sexual ao longo da vida (RP: 1,97; IC95% 1,52–2,55) (Tabela 1).

No modelo multivariado final, as variáveis associadas ao desfecho (prevalência de HIV) foram: ter 31 anos ou mais (RP: 1,76; IC95% 1,44–2,16), não estudar no momento das entrevistas (1,32; IC95% 1,01–1,73), estar desempregada (1,76; IC95% 1,10–2,80) e referência a trabalho sexual ao longo da vida (1,76; IC95% 1,35–2,29) (Tabela 2).

**Tabela 2:** Análise multivariada de fatores associados à infecção pelo HIV entre participantes do TransOdara (dezembro de 2019 a julho de 2021).

Variáveis	RPa	IC95%
Idade: 31–68 anos	1,76	[1,44–2,16]
Estudando no momento da entrevista: Não	1,32	[1,01–1,73]
Situação de trabalho: Informal	1,43	[0,97–2,12]
Situação de trabalho: Desempregada	1,76	[1,10–2,80]
Trabalho sexual ao longo da vida: Sim	1,76	[1,35–2,29]

RPa: razão de prevalência ajustada.

Desempenho comparativo cruzado usando modelos de componentes fixos.

Modelo baseado na significância estatística de análises bivariadas: identidade de gênero + orientação sexual + faixa etária + estudo [no momento da entrevista] + vínculo empregatício + sexo transacional ao longo da vida.

Modelo final baseado no modelo com melhor ajuste.

AIC = 1694,7; BIC = 1746,1; Desvio = 1674,7.

## DISCUSSÃO

Este estudo evidenciou uma prevalência elevada de HIV entre o grande número de mulheres trans e travestis recrutadas, bem como uma alta prevalência para cada um dos locais incluídos no estudo multicêntrico, variando de 26,32% a 56,08%.

Os resultados corroboram os achados anteriores tanto de estudos de base populacional como de estudos com amostras de conveniência de centros de referência^7^. Qualquer que seja o cenário e a estratégia de amostragem, as taxas de infecção pelo HIV são sempre muito elevadas e muito superiores às observadas entre as mulheres da população em geral^7^.

Uma das hipóteses para a taxa muito maior em Porto Alegre é a especificidade da epidemia no Rio Grande do Sul (RS). A taxa de detecção da AIDS no RS (24,3 por 100 mil habitantes) em 2022 foi uma vez e meia maior que a do país (16,5 por 100 mil habitantes). A taxa de mortalidade também foi superior (7,7 mortes por 100 mil habitantes) no RS à média nacional, que foi de 4,2 mortes por 100 mil habitantes. A maior taxa de mortalidade foi estimada para Porto Alegre (22,6 mortes por 100 mil habitantes), cinco vezes a taxa nacional. De acordo com o índice composto baseado nos indicadores de taxa de detecção de aids, taxa de mortalidade e primeira contagem de células CD4 dos últimos cinco anos, Porto Alegre ficou na segunda posição entre as capitais brasileiras^42^.

Numa população profundamente afetada pelo estigma e pela marginalização^6,23,43^, que vive em condições terríveis caracterizadas por habitações instáveis^43-45^, enormes dificuldades de acesso ao mercado de trabalho formal, baixos rendimentos e desemprego frequente^46-48^, essa conclusão desfavorável não é de forma alguma surpreendente. As diversas condições adversas e fatores de risco tendem a agrupar-se e a interagir frequentemente, contribuindo para padrões de vida inaceitáveis e elevada vulnerabilidade ao HIV (entre outras infecções e outras condições de saúde e sociais não avaliadas no presente estudo).

No Brasil, a prevalência do HIV nessa população é muito alta e parece improvável que seja controlada ou mesmo substancialmente melhorada, com exceção das mulheres que se beneficiam de programas de prevenção como a profilaxia pré-exposição ao HIV (PrEP)^49-52^.

Uma revisão sistemática e metanálise de estudos brasileiros implementada entre 1995 e 2016, evidenciou taxas de prevalência variando entre 14,3% (6,8–21,8) e 40,9 (35,7–46,1)^7^. Estudos mais recentes têm mostrado prevalências um pouco mais baixas, apesar dos intervalos de confiança ainda tenderem a se sobrepor, variando de 9,0% (4,2–18,2) a 31,2% (18,8–43,6) para estudos realizados em Salvador^53^ e Rio de Janeiro^15^, respectivamente.

Considerando que erros de amostragem tendem a ser ignorados (no caso de amostras de conveniência), ou são muito difíceis de estimar (no caso de estudos que utilizam as alternativas disponíveis aos estudos de probabilidade, como o RDS), é difícil distinguir tendências no sentido de um suposto declínio das taxas de infecção nos últimos anos devido a vieses secundários à inferência estatística em amostras não probabilísticas^54^.

Altas taxas de prevalência também foram demonstradas em estudos na Argentina (34,1%)^55^ e no Uruguai (21,5%)^8^. O fato de as taxas de prevalência serem quase invariavelmente superiores à estimativa única agrupada para essa população em todo o mundo (prevalência de 19,9%) é muito preocupante^7^.

No Brasil e na grande maioria dos países e contextos específicos, as mulheres trans vivem em contextos de alta vulnerabilidade e enfrentam diariamente a estigmatização e a marginalização^5,23,56-58^. O Brasil continua sendo o país de maior proporção de casos de violência contra mulheres trans, com um nível muito elevado de homicídios (em comparação aos denominadores relativamente baixos)^59,60^. Apesar dos ganhos inegáveis dos projetos específicos, essa situação lamentável exige mudanças estruturais, com o profundo compromisso de diferentes níveis do governo e da sociedade civil.

Faz-se extremamente necessário um conjunto abrangente de iniciativas coordenadas, combinando intervenções específicas (por exemplo, no domínio da saúde) com mudanças estruturais, aumentando as possibilidades de pleno acesso à educação por parte dessa população, assim como oportunidades de trabalho em diferentes tipos de empregos, especialmente aqueles atualmente longe de serem acessíveis, ou seja, empregos bem remunerados e altamente qualificados^61-65^. As elevadas taxas de participação no mercado informal, as elevadas taxas de desemprego e os rendimentos muito baixos da grande maioria dessas mulheres mostram que tais objetivos continuam a ser um sonho e não oportunidades concretas.

Ainda existe uma grande lacuna entre o que foi prometido e que foi várias vezes traduzido em leis e iniciativas não discriminatórias. É claro que tais marcos jurídicos são essenciais, mas devem ser traduzidos em iniciativas práticas e contínuas. O preconceito está profundamente arraigado e difundido. A luta para contê-lo deve ser tão presente e implacável quanto as suas raízes abertas e disfarçadas.

Não está claro se as taxas de infecção pelo HIV estão realmente diminuindo nos últimos anos ou se as supostas tendências podem ser apenas uma consequência de muitas lacunas, advertências e preconceitos. Seja qual for a verdade, a diminuição real ou percebida é muito lenta e custará muitas vidas. Pessoas já foram vítimas de homicídios e outras condições médicas graves. Esses números não são compatíveis com o que poderíamos chamar de uma sociedade humana e compassiva.

Este estudo tem limitações. O desenho transversal apresenta desafios na captura de relações temporais dentro das associações exploradas. A concepção da metodologia RDS pode introduzir vieses de seleção através de amostragem não probabilística e homofilia de rede, embora não exclua a viabilidade de realizar tais inquéritos em populações de difícil acesso. Para minimizar o viés de desenho, as análises estatísticas incorporaram a cidade como um intercepto aleatório nos modelos de regressão de Poisson de efeitos mistos. No entanto, essas limitações não nos impedem de coletar informações valiosas sobre as redes de contato da nossa amostra, alinhando-se com outros resultados da literatura.

Apesar de o Brasil ter avançado com políticas públicas (por exemplo, a Política Nacional sobre IST/AIDS e a Política Nacional de Saúde Integral LGBT), muitas barreiras permanecem, impedindo, consequentemente, a plena implementação de estratégias de prevenção do HIV. A discriminação de gênero tem sido descrita na literatura como um fator estruturante das desigualdades sociais para a população trans, particularmente no aumento da vulnerabilidade das mulheres trans e travestis à infecção pelo HIV. Este estudo reforça a necessidade de priorizar o direito ao bem-estar social digno e à qualidade de vida, bem como indica claramente a urgência de intensificar e ampliar o acesso à prevenção do HIV e à implementação de estratégias que rompam a discriminação vivida nos serviços de saúde (entre outros) e prestar serviços adequados a essa população.
