# Supplementary material for: Erratum
Source: Rev Bras Epidemiol. 2024 Oct 21;27(Suppl 1):e20240004erratum. doi: 10.1590/1980-549720240004.supl.1erratum (PMC11495134; doi:10.1590/1980-549720240004.supl.1erratum)
Supplement: Related texts

# Correction: Errata

**DOI:** 10.1590/1980-549720240004.supl.1.2erratum

No artigo **"Prevalência de infecção pelo vírus da imunodeficiência humana (HIV) em mulheres trans e travestis no Brasil: dados do estudo TransOdara"**, DOI: https://doi.org/10.1590/1980-549720240004.supl.1.2, publicado no periódico Rev Bras Epidemiol. 2024; 27(Suppl 1): e240004.supl.1

**Na página 1 foi incluído:**

Marcia Jorge Castejon^V^ http://orcid.org/0000-0002-3525-740X

^V^Instituto Adolfo Lutz, Centro de Imunologia - São Paulo, SP - Brasil.

**Na página 9, onde se lê:**

**CONTRIBUIÇÕES DOS AUTORES:** ID: conceituação, curadoria de dados, análise formal, investigação, metodologia, supervisão, escrita – primeira redação, escrita – revisão e edição. BOL: curadoria de dados, análise formal, investigação, metodologia, escrita – primeira redação, escrita – revisão e edição. LM: conceituação, curadoria de dados, análise formal, investigação, metodologia, supervisão, escrita – primeira redação, escrita – revisão e edição. FIB: conceituação, investigação, metodologia, escrita – primeira redação, escrita – revisão e edição. JCM: curadoria de dados, análise formal, metodologia, escrita – primeira redação, escrita – revisão e edição. MASMV: conceituação, curadoria de dados, análise formal, obtenção de financiamento, investigação, metodologia, administração do projeto, supervisão, escrita – primeira redação; escrita – revisão e edição.

**Leia-se:**

**CONTRIBUIÇÕES DOS AUTORES:** ID: conceituação, curadoria de dados, analise formal, investigação, metodologia, supervisa o, escrita – primeira redação, escrita – revisa o e edição. BOL: curadoria de dados, analise formal, investigação, metodologia, escrita – primeira redação, escrita – revisa o e edição. LM: conceituação, curadoria de dados, analise formal, investigação, metodologia, supervisa o, escrita – primeira redação, escrita – revisa o e edição. FIB: conceituação, investigação, metodologia, escrita – primeira redação, escrita – revisão e edição. MJC: curadoria de dados, supervisa o, analise formal, escrita – revisa o e edição. JCM: curadoria de dados, analise formal, metodologia, escrita – primeira redação, escrita – revisão e edição. MASMV: conceituação, curadoria de dados, analise formal, obtenção de financiamento, investigação, metodologia, administração do projeto, supervisa o, escrita – primeira redação; escrita – revisa o e edição.
